# Supplementary material for: Testing for pre- and post-copulatory inbreeding avoidance in the flour beetle Tribolium castaneum
Source: PLoS One. 2026 Jun 22;21(6):e0335301. doi: 10.1371/journal.pone.0335301 (PMC13286222; doi:10.1371/journal.pone.0335301)
Supplement: Attachment — Submitted filename: PONE response to reviews final_270126.docx [file pone.0335301.s001.docx]

Dear Norman,

Thank you for your work on our ms and that of the reviewers.

We have taken all of the reviewers’ comments seriously, and in the constructive spirit in which they were given, please pass on our thanks to both reviewers. We believe that we have been able to address all of their points, and that the ms is improved as a result.

We do not agree with reviewer 1’s assertion that Graur and Wool’s study from 1982 reveals a flaw in our design. Below we list the comments of the reviewers (including the two points you highlighted about references and the Graur and Wool study from 1982), and explain how we have dealt with each of them.

**Reviewer #1:** 
This manuscript explores whether Tribolium castaneum avoids inbreeding at either the pre- or post-copulatory stage and the consequences of inbreeding to progeny production. The question is biologically meaningful and the work is experimentally straightforward, though ultimately limited. Tribolium has a very long history of use in mating behavior and assortative mating / inbreeding studies. Given the voluminous literature in the system, it is not surprising to see things missed; unfortunately, there are key studies that force me to question how much generality can be gleaned from the current work.

We appreciate the reviewer’s positive comments and the work devoted to reviewing this manuscript.

Perhaps the most problematic issue I have with the design is that it has been shown that assortative mating in Tribolium can be erased by rearing two strains together. Graur and Wool (1982) used multi-choice mating designs and found positive assortative mating, but critically, the preference disappeared when unrelated beetles were co-reared before testing. That result is highly relevant because the present study shows similar assortative mating, but does not do the co-rearing, so there is no way to interpret what the apparent preference for sibs means. The authors recognize that their study cannot distinguish between familiarity and genetic relatedness driving interactions, but they do not cite (do not know?) about the earlier work showing that co-rearing can erase the kin preference. So, I am left doubting this study's interpretation of the mate preference experiments. At a minimum, this limitation to the interpretation should be explicit in the Discussion.
Graur, D., Wool, D. Dynamics and genetics of mating behavior in *Tribolium castaneum* (Coleoptera: Tenebrionidae). Behav Genet 12, 161–179 (1982), which <https://doi.org/10.1007/BF01065763>

We agree that it was an oversight not to include Graur and Wool’s work in relation to interpreting our results. We have now explicitly addressed the possibility that keeping males and female together once adult (which is what Graur and Wool did) might have led to different findings than keeping individuals in isolation (details below). Our experiment is motivated by the possibility that in the wild individuals may be able to identify they siblings either before or after mating and discriminate against them (as has been shown in other animals). In this context we would not expect adult beetles, to be co-located with a group of exclusively siblings. They would need to be in a mixed group otherwise they would not encounter both siblings and non-siblings and potentially exercise mate choice in relation to relatedness. Hence our design of keeping individuals isolated is the natural design to use to address our aims. Furthermore, in the context of this point it is worth pointing out that although Graur and Wool (1982) interpreted their work as showing that rearing individuals together erased the preference for own strain compared to if individually reared apart. In actual fact, they did not carry out an explicit test of this hypothesis. Rather, they analysed data from one experiment where individuals were reared separate from members of their own strain and found a preference for own strain. Then they conducted a separate experiment where they reared individuals together with members of their own strain and didn't find any evidence for a preference. They then compare these two results verbally and made the claim that rearing together erases the preference for own strain. This is a textbook example of an incorrect statistical inference: in order to identify whether there's an effect on preference of rearing together vs. apart you need to analyse the effect of that treatment specifically. This is easy to do using a chi-square test of independence on the 2×4 contingency table (Table VI in Graur and Wool):

BB	BD	DB	DD

Apart	24	10	12	28

Together	29	23	28	30

Test: Pearson chi-square test of independence. Null hypothesis (H₀): The distribution across BB, BD, DB, DD is the same for beetles reared together or apart χ² = 5.22 p = 0.16. This analysis shows that there was very weak, and statistically non-significant support for the hypothesis that crickets reared together or apart show a different preference. Hence Graur and Wool’s study does not actually show the effect that the reviewer focusses upon and hence we do not regard our rearing regime as a fatal flaw in our study. That being said, we do take the reviewer’s point that there is still a suggestion from Graur and Wool’s work that rearing regime might affect preference. We have discussed this point in some detail in the first paragraph of our revised methods (reference added to the revised ms reference no. 35, e.g. L147, L359).

The strain choices also need to be explained in additional detail. I suspect that both strains used here are highly inbred. The Rd mutant likely began as an isogenic female line (or nearly so). The GA1 strain has been lab-reared for several decades. These circumstances are probably unlike most (all?) Tribolium populations in nature. The Introduction and Discussion sections are written broadly, but I'm not convinced that the limitations of experimental design and strains merit general conclusions.

We agree that these strains are likely to be inbred. However, these strains are widely used in laboratory studies and have not been documented as showing inbreeding depression in any studies that we are aware of. We have some unpublished work, comparing the reindeer strain (Rd) versus other outbred stocks, e.g. KSS (used in Sales et al. 2018), the Rd strains performed equally well, perhaps due to historic purging in the lines. We agree that the ideal scenario would be that this experiment was done using wild individuals, however, a huge number of laboratory studies of insects of many species use populations that are often somewhat inbred, and we have learned a great deal from these studies (e.g. Wedell and Tregenza’s study which found post-mating inbreeding avoidance in laboratory reared field crickets using a design very similar to the one in this manuscript (Nature 1997 Vol. 386 Issue 6622 Pages 234-234).

The Rd strain carries a pronounced antennal deformity. Since antennae mediate tactile and olfactory assessment during courtship, this seems like an odd decision for a mate choice experiment since it introduces a host of questions about asymmetry in signaling between strains. The manuscript mostly assumes equivalence between strains, but no sensory or behavioral validation is presented. At a minimum, this needs to be acknowledged as a potential limitation.

The antennal deformity in the Rd strain does not seem to affect their mating performance as born out by numerous studies using this strain (e.g. Godwin 2016; Godwin et al. 2017; 2018, see L357−365). For our premating experiment only GA1 beetles were used. We have included a sentence (penultimate sentence of the 1^st^ paragraph of the methods) pointing out that the antennal deformity in the reindeer strain might influence mating performance, although we point out that there is no evidence of this. The important thing is that any such effects would be conservative in relation to our findings.

The sperm precedence data are interesting and consistent with prior sperm displacement work in Tribolium. However, the adjustment for inbred offspring survival assumes equal fertilization and egg production, yet egg counts, early larvae counts, or hatch rates are not reported. It would strengthen the study to include egg survival data or clearly acknowledge the limitation of inference based on adult numbers (Discussion line ~358–383).

We agree that genotyping fertilised eggs would be superior, we have added text to the beginning of the 3^rd^ paragraph of the discussion (L1080−1091) to acknowledge this point, and the limitation of inference based on adult numbers.

Several statistical details could be clarified: This section is not clear. In some places, the authors seem to compare totals rather than means and variances. While in other places, means and dispersion statistics are given, but not clear if these are SE or SD. Clarify the purpose/meaning of the heterogeneity tests.

Thank you – we had included a heterogeneity test to determine whether there is a broadly consistent pattern across the 27 replicates of our study. However, this is largely redundant and we agree with the thrust of the reviewer’s point, which is that the stats could be easier to follow. We have taken out this test, provided a better description of the subsequent negative binomial GLM (section 2.3, also L844−845) and made it clear which dispersion statistics are being provided.

Other terminology and clarity

Line 112: “...recessive dominant marker...” is contradictory; clarify genotype and dominance

Yes, apologies we have corrected this to visible dominant marker (L359−360)

Line ~183–188: sentence on strain interactions needs clarification (“fertilization probably or offspring viability”)

Yes, thank you, there was a typo in this sentence where ‘probability’ (L783−783) had been written as ‘probably’.

Line 242-248: There's some confusion between the figure and the text here. Fig 1A's y-axis is "contacts by males" in the figure, but the text references it as "attempted to mate". Then the next sentence points to Fig. 1 B as "mating attempts", which is consistent with what's in that figure.

Yes, this was indeed confusing. We have revisited the way we present our data, which in retrospect was not sufficiently clear. We have now combined the data from the male and female choice trials (L892−901). Instead of splitting them across 2 figures and having one panel in figure 1 which combined both trials we have a completely revised figure which we think is much clearer and we have revised the associated text.

Line 395.The Discussion ends abruptly, and it is not clear what relevance the part about thermal stress has to do with any of the rest of the work.

Yes, this is a fair point, we have extensively revised the end of the discussion and taken out the references to thermal stress.

**Reviewer #2: Major comments:**
This study investigated pre and post copulatory inbreeding avoidance in the flour beetle T. cataneum. It concluded that surprisingly males courted and mated with sibling females more so than unrelated females. Offspring proportion of females with first and second mated males indicated that the second male proportion (scored using a phenotypic marker) is not impacted by sibling versus non-sibling status (although offspring from sibling mating had lower survival).
This is a well-conducted study with simple but meaningful conclusions about lack of inbreeding avoidance in an insect system despite inbreeding cost of lower offspring viability. The elegant design combining behavioral assays and offspring scoring using a phenotypic marker is appreciated.

Thank you for the supportive remarks

Despite that, the manuscript suffers from overcomplicated interpretation and discussion. Phrasing in places is unclear. The results and their interpretation are harder to understand than they need to be, particularly as there are multiple points where statements seem to contradict each other. More relevant literature can be used throughout the manuscript. Citing general animal references is less effective than those for insect inbreeding behavior and outcomes, which are abundant, and not “rare” as authors claim based on a reference from 1996. I recommend that they review the 20 years of extensive work in this field to better contextualize their own study. Figures and figure captions need greater clarity. Some things look odd, like identical error bars.
I think the study is a strong one that is a solid novel contribution to our understanding of insect inbreeding. The readability however could be much improved. I hope the minor comments as follows can help with this:

Thank you for all your work on this, we have made changes in response to all of your suggestions

Abstract
Line 30: ? Close relatives will have close contact by default (e.g. parents and siblings). Is the idea here more to emphasize that some taxa have limited opportunity for encounters with non closely related conspecifics, such as those with poor dispersal ability?

In most species close relatives do not have any contact (ie. most insects lay eggs scattered around the environment and never see them again). It is a small minority of mainly vertebrates where parents and siblings ever meet. The idea here is to make this point, but clearly we didn’t do a good job of it. We have revised the first sentences of the abstract substantially to make it clearer (L29-33).

Line 32: Do you mean post-copulatory behavior deters remating in general or specifically against relatives

Apologies, there was a typo in this sentence which meant it didn’t really make sense, we have revised this sentence, so it should now be clear (L33-34).

Line 34: I think this is better said as “scored parentage using phenotypic markers”

Yes, good idea, we have done this (L35).

Line 37-38: Put the result about the offspring that survived to adulthood difference first, followed by how this effects the offspring proportion of doubly mated females.

Yes, good idea, we have done this (L37-39).

Line 43: It needs to be clearer that the “first in” male mad the highest fertilization proportion and that the “last in” male success declines still further over time.

The 2^nd^ male to mate has the highest fertilization proportion but this declines over time to around 50:50. We have re-written the last 2 sentences of the abstract to clarify this point. We have also clarified in the figure 3 legend that P_2_ is the proportion of offspring sired by the 2^nd^ male to mate (L41-46).

Line 49: This could be better explained, as the accumulation and expression of deleterious alleles or the loss of advantageous heterozygosity.

We have modified the text to use the reviewer’s suggested form of words (L108).

Line 51: See Leung et al. 2025 for a insect-specific review on inbreeding depression in wild and natural insect populations.

Thank you, we have added this reference

Leung, K., Beukeboom, L. W., & Zwaan, B. J. (2025). Inbreeding and outbreeding depression in wild and captive insect populations. Annual review of entomology, 70(1), 271-292.

Line 60: This section can be much more arthropod-driven. See

Metzger M, Bernstein C, Hoffmeister TS, Desouhant E (2010) Does Kin Recognition and Sib-Mating Avoidance Limit the Risk of Genetic Incompatibility in a Parasitic Wasp? PLoS ONE 5(10): e13505. <https://doi.org/10.1371/journal.pone.0013505>,

Tien, N. S. H., Massourakis, G., Sabelis, M. W., & Egas, M. (2011). Mate choice promotes inbreeding avoidance in the two-spotted spider mite. Experimental and Applied Acarology, 54(2), 119-124,

Laudani, F., Campolo, O., Latella, I., Modafferi, A., Palmeri, V., & Giunti, G. (2024). Does Hermetia illucens recognize sibling mates to avoid inbreeding depression?. Entomologia Generalis, 44(5).

We have modified this section and included all the references suggested.

Line 63: Insert “multiple” before matings

Done

Line 70: It is odd to discuss shrews and birds instead of the already extensive evidence that insect species widely employ polyandry to avoid inbreeding. See ants, bees, butterflies, flies; a meta-analysis of 122 studies is in Arnqvist, G., & Nilsson, T. (2000). The evolution of polyandry: multiple mating and female fitness in insects. Animal behaviour, 60(2), 145-164. But also the extensive evidence that inbreeding and sib-mating is common in insects. See fig wasps, gregarious parasitoids, ambrosia beetles, bed bugs, fruit flies.

We have modified this section to include more recent references on insects (L127-130).

Studies of potential precopulatory and postcopulatory inbreeding avoidance in *Drosophila melanogaster* have failed to find any evidence for it [25,26], but it has been identified in field crickets

<https://doi.org/10.1098/rspb.2003.2563>;

[https://doi.org/10.1111/jeb.12118](https://eur03.safelinks.protection.outlook.com/?url=https%3A%2F%2Fdoi.org%2F10.1111%2Fjeb.12118&data=05%7C02%7CR.Vasudeva%40exeter.ac.uk%7C13a04bd98a5e43cbdfaf08de59be435b%7C912a5d77fb984eeeaf321334d8f04a53%7C0%7C0%7C639046870870511393%7CUnknown%7CTWFpbGZsb3d8eyJFbXB0eU1hcGkiOnRydWUsIlYiOiIwLjAuMDAwMCIsIlAiOiJXaW4zMiIsIkFOIjoiTWFpbCIsIldUIjoyfQ%3D%3D%7C0%7C%7C%7C&sdata=hNqcteZAMPLBbAd%2Bbr7KQ4SnaTPKVXt2mEWXqVj9XTg%3D&reserved=0).

There is evidence for inbreeding avoidance in laboratory studies with 139 studies identified by De Boer at al., listed some lab studies, using the review in de boer et al., and also there are some refs in

<https://doi.org/10.1111/j.1365-294X.2011.05140.x>.

Also,

Liu X, Tu X, He H, Chen C, Xue F. Evidence for inbreeding depression and pre-copulatory, but not post copulatory inbreeding avoidance in the cabbage beetle Colaphellus bowringi. PloS one. 2014 Apr 9;9(4):e94389.

Line 74: Evidence for inbreeding avoidance and tolerance in invertebrates is not limited or rarely studied. The reference used to support this statement is from 1996, and there have been probably hundreds to thousands of studies on this topic in the twenty years since. Again, see Leung et al., 2025 for insects.

We have modified this section to tone down the claim about inbreeding avoidance in invertebrates being rarely studied and have included more recent references (L132-143; L135).

Line 83-96: This paragraph is a nice review of what is known about T. casteum inbreeding.

Thank you

Line 101: Here, note that the experiment focuses on related versus unrelated male mates

We do also collect data on potential male discrimination against female mates in our experiments where a single male is placed with 2 females. We have modified the sentence to be more explicit about the potential post copulatory mate choice by females (L333−335).

Line 104: Describe a “standard age”

This is an experimental practicality which isn’t needed here and just causes confusion so we’ve taken this point out.

Line 105: put “standard rearing conditions” before 30C

Done

Lines 108-113: The detail and controlled variables are appreciated here

Thank you

Line 128: Sample sizes of 30 and lower seem low.

Each of these is a trial which provides multiple contacts attempted matings and matings. Only very weak effects would be missed by a study of this size.

Line 140: Is this proportion data, or also counts? Also, the male mate choice and female mate choice data are paired, correct? Make sure this is accounted for in statistical analyses (lack of independence) and it would also make sense to have it in one methods section. Also, it seems to make more sense to have one female with two males, rather than the other way around.

These data are counts, we have made this more explicit in the revised text (section 2.1 L367 onwards). The male and female mate choice data are not paired. The comment about it making more sense to have one female with two males indicates that we were not clear enough in explaining the design (we had both one male with 2 females and 2 females with one male). We have revised our description of these trials including following the suggestion of not having separate sections for the 2 types of trial. We have also revised our presentation of the results (combining figures 1 and 2) which we think is a lot clearer.

Figure 1: This figure and its caption needs improvements. First is to note that it is data for the GA1 wildtype strain. Markers of (non)significant difference are needed among the treatments. Labels on 1B is confusing because they are relational but unclear. How can a male mate with a female as a “non sib” if the female type itself is “sib” etc.
Figure 2: It is not clear if this data is independent from Figure 1 (if so, is this figure redundant?). The square, diamond, and circle markers do not seem needed here, as there is no overlap among them. The Rd strain was not mentioned in this set of experiment(s) in methods. Markers of (non)significant difference are still needed.

In retrospect we agree that figures 1 and 2 were not nearly clear enough (L533 onwards). We have completely revised these figures so that we now have a single figure which includes all the data from the two types of trial (female choice and male choice). We have combined contacts ‘initiated by the male’ and those ‘initiated by the female’ which were in figure 2. The difference between these two types of contact is not extremely clear and there are no differences between the categories, and in the trials with 2 males and a single female all contacts were initiated by males, so it was hard to compare contacts data between the 2 types of trial.

Line 179: Briefly describe the study design of Tregenza and Wedell.

Done, we changed the following sentence to avoid repetition and to increase clarity.

Line 202-206: Good design

Thank you

Line 209: Explain why the males were mated to a stock female the day before

Done – we did this to check the males were willing to mate with a female when presented with one (section 2.2).

Line 212-216: To understand this experiment better, more information about the full life cycle would be helpful in the materials and methods section would be helpful. E.g. what is the range of how long a T. casteum female lives and reproduces if fed?

Good idea, we have added a sentence to the beginning of the methods that summarises the life cycle of *Tribolium castaneum* (section 2, L336 onwards upto L340)*.*

Line 216: Clearer explanation here that this be done to ID the father (whether offspring is P1 or P2). Explain why there was a 1-week reproductive experiment for most females, but only a “lifetime offspring paternity test” for N=17? I expect just limitations of time, but say so if so.

Done

Line 233: What are these error distributions? Line 235: Sentence fragment

These are the distributions set out in the next sentence, we have revised this whole paragraph for clarity and to get rid of the sentence fragment mentioned above.

Line 244: Tracked individual data (means and standard error, with replicate number noted) would be more informative than these pooled numbers.

Much more detail about the data is provided in the figures, our intention here was just to give a very easily understood single figure summary.

Figure 3: The error bars in A are identical. Is this a mistake? And indicate what they signify. Standard error or deviation? Note in the caption once again what P2 is, for clarity. The SS- NS etc is confusing notation. Both in caption and the figure labels, make clear that it indicates the order of males for the doubly mated females. Something like S1S2, S1N2, and write out what this means in the caption and use consistently in MS.

These error bars are very similar (in the revised ms, it is figure 2), but they are not identical: there is more potential error around the N-N mean than the other three, and those error bars are slightly bigger. Error bars are standard errors, we have added this to the legend. We use the S-S notation following other studies including the one that our design is based on which use this terminology, we have modified the figure legend to explain the N and S designations and avoid the confusing switching between ‘sib’ and ‘related’. We have also revised the 2^nd^ paragraph under ‘post-mating inbreeding avoidance’ to clarify the terminology.

Line 273: This could be written clearer. The idea is, that the observation period is long to prevent limiting the possible number of mating attempts.

We have rewritten this sentence to clarify our meaning which is that matings are short compared to the observation period, so we don’t expect a relationship between the duration of matings and the number of matings arising because of a shortage of time (L954-958).

Line 294: The datapoint for SS seems higher than the others. Write the sample size N for each treatment

10 is indeed higher than 4, but as in the subsequent test (χ^2^ = 5.9, P = 0.12) there is no statistical support for this being something we can comment on. We have added the sample sizes to the sentence as suggested (L974-983).

Line 306: Unclear if this data is average for individual (is there a standard error)? The statistical test is missing. Also, this P2 data is “raw” but why not put it in a single section with the adjusted P2 results? This seems a distinct category than the post-mating inbreeding avoidance data of the section it is currently in.

Apologies for lack of clarity here. We think these data will be of interest to readers as they represent P_2_ values over a longer time frame than most studies. We have clarified that these are means and standard errors (L985-989).

Line 322: “giving a value of 1.42”

Done

Discussion is general is very muddled. I am not sure at all what the conclusions are getting at, for what I thought was a simple dataset indicating preferential male mating for siblings despite the cost of lower survival of inbred offspring to adulthood (and mating order of multiple males not impacting offspring proportion). I think authors should try to couch their results in comparisons to other insect inbreeding studies.

We have revised the discussion to try to make it clearer including addressing the specific points below (section 4).

Line 330: Clarify the “sex ratio” treatment statement: note that it is the two females, one males, and vice versa treatments.

We have revised the sentence so that it reads “trials with two males and one female and with one female and two males” (L1058-1068)

Line 340: The ideas in this paragraph are not clear. First it details that “male initiation behavior” is the majority. Then it talks about initiation being exclusive to males. But in the introduction, it was mentioned that female initiation behavior also occurs, but no data is reported for it here. What is the main idea? How does it all connect? I think it has to do with females simply having lower initiation rates, but that males indiscriminately initiate with males and females.

In retrospect we agree with the reviewer that this paragraph is not very clear. I think we got interested in the idea that we might have some insights into the role of the two sexes in mate choice decisions, but really we don’t have very good data on this and this paragraph is a distraction, we have deleted the paragraph to focus on the point the reviewer makes about this being a simple dataset indicating preferential male mating for siblings despite the cost of lower survival of inbred offspring to adulthood (and mating order of multiple males not impacting offspring proportion) (L1058-1075).

Line 344: How can males recognize their sisters (specific females) when the previous paragraph states they cannot even tell the difference between males and females?

Yes, this is a fair point (although over-simplifying somewhat); we have removed the previous paragraph which was too speculative (L1058-1075).

Line 346: I do not understand the idea of this sentence. Why would both preferential sib mating, an inbreeding depression be expected? These are intuitively oppositional selective pressures.

In these sentences we were attempting to both highlight the fact that mating patterns suggest that kin-recognition does occur, and then to discuss the potential mechanisms. However, it clearly wasn’t clear enough, we have revised the paragraph to separate these points (L1070-1075).

Line 353: This is a contradictory conclusion. Authors’s data shows inbreeding depression of lower offspring survival to adulthood with sibling matings. Here it says that there are relatively low costs of inbreeding. I can see this making sense on a population level, if the purging of inbred offspring is beneficial, but not sure what is meant.

This conclusion appears contradictory, but it stems from the fact that an allele which encourages females to mate with their brothers (who have a 50% chance of carrying that allele) will tend to increase in frequency. The basis for this assertion is provided in the references we have cited and we have added the short explanation above (L1058-1068).

Line 349: “cannot distinguish between learned familiarity and genetic similarity”. This is incredibly vague and very confusing, and it does not need to be when authors can cite the many studies that examine mechanisms for insect.

We have taken out this phrase and made the point in a different construction of the paragraph (L1070-1075).

Line 382: Saying “inbreeding avoidance are taxonomically widespread and have been shown to be the rule among vertebrates” is in direct contradiction to what was previously said by the authors about inbreeding avoidance being rare using reference de Boer et al. [47]: de Boer RA, Vega-Trejo R, Kotrschal A, Fitzpatrick JL. Meta-analytic evidence that animals rarely avoid inbreeding. Nat Ecol Evol. 2021 Jul;5(7):949-964. doi: 10.1038/s41559-021-01453-9. Epub 2021 May 3. PMID: 33941905.

Yes, the reviewer is correct our discussion was not sufficiently up-to-date. We have extensively revised it to make the points raised (L1077-1201).

Line 386: Iridomymex humile is a long retired synonym of the Argentine ant Linepithema humile. Also, this is a bad species to try to make a general comparison to a beetle for many reasons. One, there are only a few reproductives, so population dynamics will differ. Two, it has complementary sex determination with extremely high inbreeding cost due to a single gene that causes sterilization if homozygous. There are many studies on the complicated implications of its inbreeding, including as an invasive where extreme genetic load of bottlenecked populations can be weighed against reduced intraspecific aggression for e.g. supercolony formation (this is too complicated to get into here, but for more info on CSD inbreeding, see Zayed and Parker 2005, Leung and van der Meulen 2022).

Thank you, we have removed this reference and added references to other more recent studies (L1209-1211).

Line 388: Miswritten, “have similar sibling and non-sibling mating rates”. Of course, for ants, this only applies to queens, and polyandry to reduce impacts of sib mating is possible.

We have used the text suggested, we have not added the comment about it only applying to queens as readers will be aware of this (L1211).

Line 391: What are these other fitness costs? List them or give examples.

We have now included this information (L1214-1218). Males experiencing greater inbreeding load suffered from shorter sperm, smaller testes volumes, smaller body sizes and reduced lifespan compared to males that were under control non-inbred conditions. Reproductive output was affected as well, but that value was a composite of both male and female inbreeding status (as inbred females were paired with inbred males).

Line 397: The thermal idea comes out of nowhere. Is it supposed to connect to climate change and inbreeding increase? Why?

We have taken out this sentence.

References: Formatting is inconsistent (dois for some, not forothers; capitalization varies; italics missing for species, etc).

Apologies. We have now checked our references (References section, L1318 onwards).
