# Supplementary material for: Testing for pre- and post-copulatory inbreeding avoidance in the flour beetle Tribolium castaneum
Source: PLoS One. 2026 Jun 22;21(6):e0335301. doi: 10.1371/journal.pone.0335301 (PMC13286222; doi:10.1371/journal.pone.0335301)
Supplement: Attachment — Submitted filename: PONE 2nd response to reviewers.docx [file pone.0335301.s002.docx]

Dear Norman,

Thank you for your positive feedback on our revised manuscript and that of the reviewers. We have followed the reviewers most recent suggestions in relation to the ms as detailed below.

Reviewer #1: I appreciate the authors’ extensive revisions and the considerable effort that went into improving the manuscript. The paper is clearer and better contextualized than the original submission, particularly with respect to the revised figures, the expanded insect literature, and the more focused Discussion. I also appreciate the authors’ willingness to engage seriously with my initial comments.

Thank you.

That said, I remain unconvinced by the authors’ treatment of the Graur and Wool (1982) issue, which I still view as central to interpretation of the assortative mating results. My concern was not simply whether rearing treatment and mating outcome are statistically associated in a pooled 2×4 contingency table. Rather, the biologically relevant observation from Graur and Wool is that assortative mating was observed under one rearing regime (“reared apart”) and disappeared under the other (“reared together”). The present study effectively reproduces only the “reared apart” condition. Therefore, the key interpretive limitation remains: it is unclear whether the observed sibling preference reflects kin recognition, familiarity effects, or an artifact of the isolation-rearing design. The lack of significance in the pooled association test does not negate the observation that assortative mating was present in one treatment and absent in the other. As written, the response reads as though the authors are attempting to statistically dismiss a biologically meaningful pattern rather than acknowledge it as an important caveat for the interpretation of their own findings.

I also do not find the authors’ argument persuasive that isolation is the “natural” design because beetles in nature would encounter mixed-relatedness groups. If anything, this seems to strengthen the rationale for testing beetles under mixed-rearing conditions rather than isolated rearing. If the goal is to infer mate choice under natural social conditions, rearing individuals in complete isolation may remove developmental and social cues that are themselves biologically important to mate recognition. Thus, I still think the manuscript should frame the assortative mating result much more cautiously and explicitly acknowledge the limitations imposed by the rearing design.

The reason for our statistical analysis was to determine whether the result in Graur and Wool is likely to be biologically meaningful or could simply be a result of chance. That analysis shows that there may actually be no effect of rearing alone or apart, but it was not designed to ‘get us off the hook’ in relation to this point. We take the reviewer’s point that even if there is only very weak evidence (or indeed, no evidence) that rearing apart as opposed to in a group has an effect, it remains possible that it does. We have added a new paragraph to the discussion where we discuss this point. We have added new references to studies of other insects that show the potential for the type of effect that the reviewer is referring to. We have borrowed the reviewer’s specific wording that “rearing individuals in complete isolation may remove developmental and social cues that are themselves biologically important to mate recognition” **(L361-362)**– we agree with the thrust of this point. We think with this new discussion readers will be fully aware of the issues the reviewer is referring to.

There is also a concerning error in the rebuttal text immediately following the contingency-table discussion, where Tribolium are mistakenly referred to as “crickets.” While obviously minor in isolation, it contributes to a broader impression of the work.

Yes, apologies for this careless typo in our letter.

I continue to think the study contains meaningful results that are consistent with previous work in the system especially regarding offspring viability and sperm precedence dynamics, and the manuscript is improved overall. However, I still believe the conclusions regarding assortative mating need to be substantially tempered. The final two paragraphs of their conclusions rest on having shown that Tribolium demonstrate positive assortative mating, but I don't think their designs allows them to make this conclusion outside the very narrow scope of their rearing/breeding design, and consequently the attempts to explain it are moot.

We have revised our text so that we repeatedly point out that our conclusions can only be very confidently ascribed to the rearing situation that we employed (e.g., **L363-365**).

Reviewer #2: The manuscript is much improved in its use of updated literature and corrections of various small errors, unclear language, and unsubstantiated discussion points (thermal stress). It is to be commended for clear and effective counter points for all concerns across all reviewers. I give minor comments below, but otherwise recommend acceptance.

Thank you

Line 30: unclear what by “insects without parental care,” does not connect to the idea of with multiple mating

Yes, fair point we have taken out that clause as it either needs a lot more explanation or needs to be left out, which makes more sense.

Line 34: Better phrased as “direct observation of breeding behavior and subsequent scoring of offspring parentage using phenotypic markers”

Thank you we have used the suggested text

Line 37: Offspring of unrelated parents?

Yes we have revised this sentence in line with this suggestion.

Line 38: Not clear what “raw” count is

Yes, good point, we have removed the confusing reference to ‘raw count’.

Line 40: Fertilisation success of male with related and unrelated

We have revised this sentence

Line 42: This result is unclear. Did total number of offspring even out between 1st and 2nd male over the 5 weeks, or did new offspring after week 5 have equal percentage paternity?

This is a very good point; we have revised the text to clarify that it is the latter scenario.

Line 49: Homozygosity across the whole genome, one gene, or?

We have added ‘across the genome’.

Line 59: Make clear that this is for mate choice.

Done

Line 75: Studies in Drosophila failed to find any evidence for potential pre-or post-copulatory inbreeding avoidance.

Done

Line 97: Missing word after “P2”? What do the more attractive males have more of?

We have removed the term P2 from the abstract in relation to the suggestion made below.

Line 105: “mated to”

Done

Line 107: Italicize Tribolium castaneum

Done

Line 265: Here the meaning of P2 becomes clear, but please make it clear in abstract and earlier. P2 instinctively sounds like a parental generation notation.

Apologies – it is a good point that the term P2 will not be familiar to all readers. We have removed it from the abstract and explain it fully the first time we use it in the revised manuscript (**L98-99**).

Figure 2B: It is still not clear to me what is meant about the 2nd male effect resulting in equal performance between the sib and non-sib. From this figure, I think the conclusion is drawn from pooling data of each type of treatment across data points for multiple weeks, not a reversed trend at some point. Please make this clear in wording throughout.

It is not the 2^nd^ male effect that results in equal performance, it is adjusting for the fact that offspring from sibling males are less likely to survive (developmental failures) so their fertilisation success is underestimated if we just use the raw number of offspring they sire. It is clear that our text was not clear enough on this point, we have revised the figure legend to clarify this point where it is most important (**L265-250**).

Line 340: I think the result of preferential sibling mating is significant, but that the effect size isn’t very big (with the exception of the female mate choice for mating attempts? But the difference is something like 2 vs 4, and there is a lot of scatter for the sib mating data; Figure 1B). I think it better to be transparent about this, as it is still of great importance and strong statement that there isn’t major inbreeding avoidance. I am not sure if the authors want to be emphatic about evidence for sibling preference.

Yes, this is a fair point. We have revised the text so that it reads that “Males placed with a sibling and a non-sibling female attempted to mate with their sisters and actually succeeded in mating with them slightly more often than unrelated females.” (**L345-347**) Adding the word ‘slightly’ is a small addition, but we think it does convey the lack of being emphatic that the reviewer is requesting.

Line 349: In the same vein, do authors want to take such a soft stance on inbreeding depression? In figure 1D, there is definitely a cost to offspring viability with sib mating that doesn’t seem that small (~25%). Coming to a paradoxical conclusion of some sibling preference/no sibling avoidance despite offspring depression, would also be valid. There could be hidden benefits (e.g. with the authors’ suggestion of inclusive fitness), but it could also just be maladaptive.

We do point out the costs of inbreeding are large. However, as shown in the models we cite, the inclusive fitness benefits of sib mating can be large, and this is reflected in our text (**L355-369**). It could simply be maladaptive, but we feel that this is something that readers will be aware of, and adding a caveat of that type is more likely to puzzle readers than to enlighten them.

Line 357: Not sure what is meant by highly polymorphic genes. Siblings would have the same alleles and perceive them via phenotype? Perhaps the idea of phenotype matching here makes sense, as in

Mathieu Lihoreau, Cédric Zimmer, Colette Rivault, Kin recognition and incest avoidance in a group-living insect, Behavioral Ecology, Volume 18, Issue 5, September 2007, Pages 880–887, https://doi.org/10.1093/beheco/arm046

Or speculation on recognizing olfactory cues e.g. as with sibling recognition in social species

Couto, A., Marty, S., Dawson, E.H., d'Ettorre, P., Sandoz, J.-C. and Montgomery, S.H. (2023), Evolution of the neuronal substrate for kin recognition in social Hymenoptera. Biol Rev, 98: 2226-2242. <https://doi.org/10.1111/brv.13003>

Thank you – we clarified our point (which is as the reviewer suggests) and added the 1^st^ reference suggested above.

Line 390-392: There is a rough transition here of going from saying inbreeding avoidance isn’t as prevalent as previously thought into a big inbreeding avoidance example. Needs a phrase like: “There are clear cases where inbreeding avoidance is employed; for example, crickets”… and then it switches over naturally to the inbreeding prone species. I think there are also good examples from bedbugs and parasitoids where sib mating is the rule.

Thank you we included the suggested text in the revised manuscript.
