# Supplementary material for: Testing for pre- and post-copulatory inbreeding avoidance in the flour beetle Tribolium castaneum
Source: PLoS One. 2026 Jun 22;21(6):e0335301. doi: 10.1371/journal.pone.0335301 (PMC13286222; doi:10.1371/journal.pone.0335301)
Supplement: Peer review history

# Peer review: Decision Letter 0

**DOI:** 10.1371/journal.pone.0335301.r001

**Authors:** Norman Johnson

24 Nov 2025

PONE-D-25-54427

Testing for pre- and post-copulatory inbreeding avoidance in the flour beetle Tribolium castaneum

PLOS ONE

Dear Dr. Vasudeva,

Thank you for submitting your manuscript to PLOS ONE. After careful consideration, we feel that it has merit but does not fully meet PLOS ONE’s publication criteria as it currently stands. Therefore, we invite you to submit a revised version of the manuscript that addresses the points raised during the review process.

Both reviewers had substantial comments / concerns about the manuscript.

Reviewer 1 brought up an important point: “….assortative mating in Tribolium can be erased by rearing two strains together. Graur and Wool (1982) used multi-choice mating designs and found positive assortative mating, but critically, the preference disappeared when unrelated beetles were co-reared before testing.”

I think the authors should either repeat the experiments with the strains raised together or justify why this is unnecessary.

Reviewer 2 noted that the references are inadequate. I agree. This is easily fixed.

Please submit your revised manuscript by Jan 08 2026 11:59PM. If you will need more time than this to complete your revisions, please reply to this message or contact the journal office at plosone@plos.org. When you're ready to submit your revision, log on to https://www.editorialmanager.com/pone/ and select the 'Submissions Needing Revision' folder to locate your manuscript file.

Please include the following items when submitting your revised manuscript:

- A rebuttal letter that responds to each point raised by the academic editor and reviewer(s). You should upload this letter as a separate file labeled 'Response to Reviewers'.
- A marked-up copy of your manuscript that highlights changes made to the original version. You should upload this as a separate file labeled 'Revised Manuscript with Track Changes'.
- An unmarked version of your revised paper without tracked changes. You should upload this as a separate file labeled 'Manuscript'.

If you would like to make changes to your financial disclosure, please include your updated statement in your cover letter. Guidelines for resubmitting your figure files are available below the reviewer comments at the end of this letter.

If applicable, we recommend that you deposit your laboratory protocols in protocols.io to enhance the reproducibility of your results. Protocols.io assigns your protocol its own identifier (DOI) so that it can be cited independently in the future. For instructions see: https://journals.plos.org/plosone/s/submission-guidelines#loc-laboratory-protocols. Additionally, PLOS ONE offers an option for publishing peer-reviewed Lab Protocol articles, which describe protocols hosted on protocols.io. Read more information on sharing protocols at https://plos.org/protocols?utm_medium=editorial-email&utm_source=authorletters&utm_campaign=protocols.

We look forward to receiving your revised manuscript.

Kind regards,

Norman Johnson

Academic Editor

PLOS ONE

Journal Requirements:

When submitting your revision, we need you to address these additional requirements.

1. Please ensure that your manuscript meets PLOS ONE's style requirements, including those for file naming. The PLOS ONE style templates can be found at

https://journals.plos.org/plosone/s/file?id=wjVg/PLOSOne_formatting_sample_main_body.pdf and

https://journals.plos.org/plosone/s/file?id=ba62/PLOSOne_formatting_sample_title_authors_affiliations.pdf

2. Please note that your Data Availability Statement is currently missing the repository name. If your manuscript is accepted for publication, you will be asked to provide these details on a very short timeline. We therefore suggest that you provide this information now, though we will not hold up the peer review process if you are unable.

3. If the reviewer comments include a recommendation to cite specific previously published works, please review and evaluate these publications to determine whether they are relevant and should be cited. There is no requirement to cite these works unless the editor has indicated otherwise.

**Additional Editor Comments:**

The references section regarding insect inbreeding needs to be expanded.

[Note: HTML markup is below. Please do not edit.]

Reviewers' comments:

Reviewer's Responses to Questions

**Comments to the Author**

1. Is the manuscript technically sound, and do the data support the conclusions?

The manuscript must describe a technically sound piece of scientific research with data that supports the conclusions. Experiments must have been conducted rigorously, with appropriate controls, replication, and sample sizes. The conclusions must be drawn appropriately based on the data presented.

Reviewer #1: Partly

Reviewer #2: Partly

**********

2. Has the statistical analysis been performed appropriately and rigorously?

Reviewer #1: No

Reviewer #2: I Don't Know

**********

3. Have the authors made all data underlying the findings in their manuscript fully available?

The PLOS Data policy requires authors to make all data underlying the findings described in their manuscript fully available without restriction, with rare exception (please refer to the Data Availability Statement in the manuscript PDF file). The data should be provided as part of the manuscript or its supporting information, or deposited to a public repository. For example, in addition to summary statistics, the data points behind means, medians and variance measures should be available. If there are restrictions on publicly sharing data—e.g. participant privacy or use of data from a third party—those must be specified.

Reviewer #1: Yes

Reviewer #2: Yes

**********

4. Is the manuscript presented in an intelligible fashion and written in standard English?

PLOS ONE does not copyedit accepted manuscripts, so the language in submitted articles must be clear, correct, and unambiguous. Any typographical or grammatical errors should be corrected at revision, so please note any specific errors here.

Reviewer #1: Yes

Reviewer #2: Yes

**********

5. Review Comments to the Author

Please use the space provided to explain your answers to the questions above. You may also include additional comments for the author, including concerns about dual publication, research ethics, or publication ethics. (Please upload your review as an attachment if it exceeds 20,000 characters)

Reviewer #1: This manuscript explores whether Tribolium castaneum avoids inbreeding at either the pre- or post-copulatory stage and the consequences of inbreeding to progeny production. The question is biologically meaningful and the work is experimentally straightforward, though ultimately limited. Tribolium has a very long history of use in mating behavior and assortative mating / inbreeding studies. Given the voluminous literature in the system, it is not surprising to see things missed; unfortunately, there are key studies that force me to question how much generality can be gleaned from the current work.

Perhaps the most problematic issue I have with the design is that it has been shown that assortative mating in Tribolium can be erased by rearing two strains together. Graur and Wool (1982) used multi-choice mating designs and found positive assortative mating, but critically, the preference disappeared when unrelated beetles were co-reared before testing. That result is highly relevant because the present study shows similar assortative mating, but does not do the co-rearing, so there is no way to interpret what the apparent preference for sibs means. The authors recognize that their study cannot distinguish between familiarity and genetic relatedness driving interactions, but they do not cite (do not know?) about the earlier work showing that co-rearing can erase the kin preference. So, I am left doubting this study's interpretation of the mate preference experiments. At a minimum, this limitation to the interpretation should be explicit in the Discussion.

Graur, D., Wool, D. Dynamics and genetics of mating behavior in Tribolium castaneum (Coleoptera: Tenebrionidae). Behav Genet 12, 161–179 (1982), which https://doi.org/10.1007/BF01065763

The strain choices also need to be explained in additional detail. I suspect that both strains used here are highly inbred. The Rd mutant likely began as an isogenic female line (or nearly so). The GA1 strain has been lab-reared for several decades. These circumstances are probably unlike most (all?) Tribolium populations in nature. The Introduction and Discussion sections are written broadly, but I'm not convinced that the limitations of experimental design and strains merit general conclusions.

The Rd strain carries a pronounced antennal deformity. Since antennae mediate tactile and olfactory assessment during courtship, this seems like an odd decision for a mate choice experiment since it introduces a host of questions about asymmetry in signaling between strains. The manuscript mostly assumes equivalence between strains, but no sensory or behavioral validation is presented. At a minimum, this needs to be acknowledged as a potential limitation.

For the premating experiment, were only GA1 beetles used? Lines 119-134 only mention GA1, but the preceding paragraph introduces both strains, and later, it seems non-sib = beetle from the other strain, so I'm not certain.

The sperm precedence data are interesting and consistent with prior sperm displacement work in Tribolium. However, the adjustment for inbred offspring survival assumes equal fertilization and egg production, yet egg counts, early larvae counts, or hatch rates are not reported. It would strengthen the study to include egg survival data or clearly acknowledge the limitation of inference based on adult numbers (Discussion line ~358–383).

Several statistical details could be clarified: This section is not clear. In some places, the authors seem to compare totals rather than means and variances. While in other places, means and dispersion statistics are given, but not clear if these are SE or SD. Clarify the purpose/meaning of the heterogeneity tests.

Other terminology and clarity

Line 112: “...recessive dominant marker...” is contradictory; clarify genotype and dominance

Line ~183–188: sentence on strain interactions needs clarification (“fertilization probably or offspring viability”)

Line 242-248: There's some confusion between the figure and the text here. Fig 1A's y-axis is "contacts by males" in the figure, but the text references it as "attempted to mate". Then the next sentence points to Fig. 1 B as "mating attempts", which is consistent with what's in that figure.

Line 395ff.The Discussion ends abruptly, and it is not clear what relevance the part about thermal stress has to do with any of the rest of the work.

Reviewer #2: Major comments:

This study investigated pre and post copulatory inbreeding avoidance in the flour beetle T. cataneum. It concluded that surprisingly males courted and mated with sibling females more so than unrelated females. Offspring proportion of females with first and second mated males indicated that the second male proportion (scored using a phenotypic marker) is not impacted by sibling versus non-sibling status (although offspring from sibling mating had lower survival).

This is a well-conducted study with simple but meaningful conclusions about lack of inbreeding avoidance in an insect system despite inbreeding cost of lower offspring viability. The elegant design combining behavioral assays and offspring scoring using a phenotypic marker is appreciated.

Despite that, the manuscript suffers from overcomplicated interpretation and discussion. Phrasing in places is unclear. The results and their interpretation are harder to understand than they need to be, particularly as there are multiple points where statements seem to contradict each other. More relevant literature can be used throughout the manuscript. Citing general animal references is less effective than those for insect inbreeding behavior and outcomes, which are abundant, and not “rare” as authors claim based on a reference from 1996. I recommend that they review the 20 years of extensive work in this field to better contextualize their own study. Figures and figure captions need greater clarity. Some things look odd, like identical error bars.

I think the study is a strong one that is a solid novel contribution to our understanding of insect inbreeding. The readability however could be much improved. I hope the minor comments as follows can help with this:

Abstract

Line 30: ? Close relatives will have close contact by default (e.g. parents and siblings). Is the idea here more to emphasize that some taxa have limited opportunity for encounters with non closely related conspecifics, such as those with poor dispersal ability?

Line 32: Do you mean post-copulatory behavior deters remating in general or specifically against relatives

Line 34: I think this is better said as “scored parentage using phenotypic markers”

Line 37-38: Put the result about the offspring that survived to adulthood difference first, followed by how this effects the offspring proportion of doubly mated females.

Line 43: It needs to be clearer that the “first in” male mad the highest fertilization proportion and that the “last in” male success declines still further over time.

Line 49: This could be better explained, as the accumulation and expression of deleterious alleles or the loss of advantageous heterozygosity.

Line 51: See Leung et al. 2025 for a insect-specific review on inbreeding depression in wild and natural insect populations. Leung, K., Beukeboom, L. W., & Zwaan, B. J. (2025). Inbreeding and outbreeding depression in wild and captive insect populations. Annual review of entomology, 70(1), 271-292.

Line 60: This section can be much more arthropod-driven. See Metzger M, Bernstein C, Hoffmeister TS, Desouhant E (2010) Does Kin Recognition and Sib-Mating Avoidance Limit the Risk of Genetic Incompatibility in a Parasitic Wasp? PLoS ONE 5(10): e13505. https://doi.org/10.1371/journal.pone.0013505, Tien, N. S. H., Massourakis, G., Sabelis, M. W., & Egas, M. (2011). Mate choice promotes inbreeding avoidance in the two-spotted spider mite. Experimental and Applied Acarology, 54(2), 119-124, Laudani, F., Campolo, O., Latella, I., Modafferi, A., Palmeri, V., & Giunti, G. (2024). Does Hermetia illucens recognize sibling mates to avoid inbreeding depression?. Entomologia Generalis, 44(5).

Line 63: Insert “multiple” before matings

Line 70: It is odd to discuss shrews and birds instead of the already extensive evidence that insect species widely employ polyandry to avoid inbreeding. See ants, bees, butterflies, flies; a meta-analysis of 122 studies is in Arnqvist, G., & Nilsson, T. (2000). The evolution of polyandry: multiple mating and female fitness in insects. Animal behaviour, 60(2), 145-164. But also the extensive evidence that inbreeding and sib-mating is common in insects. See fig wasps, gregarious parasitoids, ambrosia beetles, bed bugs, fruit flies.

Line 74: Evidence for inbreeding avoidance and tolerance in invertebrates is not limited or rarely studied. The reference used to support this statement is from 1996, and there have been probably hundreds to thousands of studies on this topic in the twenty years since. Again, see Leung et al., 2025 for insects.

Line 83-96: This paragraph is a nice review of what is known about T. casteum inbreeding.

Line 101: Here, note that the experiment focuses on related versus unrelated male mates

Line 104: Describe a “standard age”

Line 105: put “standard rearing conditions” before 30C

Lines 108-113: The detail and controlled variables are appreciated here

Line 128: Sample sizes of 30 and lower seem low.

Line 140: Is this proportion data, or also counts? Also, the male mate choice and female mate choice data are paired, correct? Make sure this is accounted for in statistical analyses (lack of independence) and it would also make sense to have it in one methods section. Also, it seems to make more sense to have one female with two males, rather than the other way around.

Figure 1: This figure and its caption needs improvements. First is to note that it is data for the GA1 wildtype strain. Markers of (non)significant difference are needed among the treatments. Labels on 1B is confusing because they are relational but unclear. How can a male mate with a female as a “non sib” if the female type itself is “sib” etc.

Figure 2: It is not clear if this data is independent from Figure 1 (if so, is this figure redundant?). The square, diamond, and circle markers do not seem needed here, as there is no overlap among them. The Rd strain was not mentioned in this set of experiment(s) in methods. Markers of (non)significant difference are still needed.

Line 179: Briefly describe the study design of Tregenza and Wedell.

Line 202-206: Good design

Line 209: Explain why the males were mated to a stock female the day before

Line 212-216: To understand this experiment better, more information about the full life cycle would be helpful in the materials and methods section would be helpful. E.g. what is the range of how long a T. casteum female lives and reproduces if fed?

Line 216: Clearer explanation here that this be done to ID the father (whether offspring is P1 or P2). Explain why there was a 1-week reproductive experiment for most females, but only a “lifetime offspring paternity test” for N=17? I expect just limitations of time, but say so if so.

Line 233: What are these error distributions?

Line 235: Sentence fragment

Line 244: Tracked individual data (means and standard error, with replicate number noted) would be more informative than these pooled numbers

Figure 3: The error bars in A are identical. Is this a mistake? And indicate what they signify. Standard error or deviation? Note in the caption once again what P2 is, for clarity. The SS- NS etc is confusing notation. Both in caption and the figure labels, make clear that it indicates the order of males for the doubly mated females. Something like S1S2, S1N2, and write out what this means in the caption and use consistently in MS

Line 273: This could be written clearer. The idea is, that the observation period is long to prevent limiting the possible number of mating attempts.

Line 294: The datapoint for SS seems higher than the others. Write the sample size N for each treatment

Line 306: Unclear if this data is average for individual (is there a standard error)? The statistical test is missing. Also, this P2 data is “raw” but why not put it in a single section with the adjusted P2 results? This seems a distinct category than the post-mating inbreeding avoidance data of the section it is currently in.

Line 322: “giving a value of 1.42”

Discussion is general is very muddled. I am not sure at all what the conclusions are getting at, for what I thought was a simple dataset indicating preferential male mating for siblings despite the cost of lower survival of inbred offspring to adulthood (and mating order of multiple males not impacting offspring proportion). I think authors should try to couch their results in comparisons to other insect inbreeding studies.

Line 330: Clarify the “sex ratio” treatment statement: note that it is the two females, one males, and vice versa treatments.

Line 340: The ideas in this paragraph are not clear. First it details that “male initiation behavior” is the majority. Then it talks about initiation being exclusive to males. But in the introduction, it was mentioned that female initiation behavior also occurs, but no data is reported for it here. What is the main idea? How does it all connect? I think it has to do with females simply having lower initiation rates, but that males indiscriminately initiate with males and females

Line 344: How can males recognize their sisters (specific females) when the previous paragraph states they cannot even tell the difference between males and females?

Line 346: I do not understand the idea of this sentence. Why would both preferential sib mating, an inbreeding depression be expected? These are intuitively oppositional selective pressures.

Line 353: This is a contradictory conclusion. Authors’s data shows inbreeding depression of lower offspring survival to adulthood with sibling matings. Here it says that there are relatively low costs of inbreeding. I can see this making sense on a population level, if the purging of inbred offspring is beneficial, but not sure what is meant.

Line 349: “cannot distinguish between learned familiarity and genetic similarity”. This is incredibly vague and very confusing, and it does not need to be when authors can cite the many studies that examine mechanisms for insect recognition/preference/discrimination against close relatives in mating. They could think upon chemosensory cues such as similarity/dissimilarity of CHC profiles, mate guarding siblings as mates in the same natal patch, dispersal immediately following eclosion/nuptial flights, etc.

Line 382: Saying “inbreeding avoidance are taxonomically widespread and have been shown to be the rule among vertebrates” is in direct contradiction to what was previously said by the authors about inbreeding avoidance being rare using reference de Boer et al. [47]: de Boer RA, Vega-Trejo R, Kotrschal A, Fitzpatrick JL. Meta-analytic evidence that animals rarely avoid inbreeding. Nat Ecol Evol. 2021 Jul;5(7):949-964. doi: 10.1038/s41559-021-01453-9. Epub 2021 May 3. PMID: 33941905.

Line 386: Iridomymex humile is a long retired synonym of the Argentine ant Linepithema humile. Also, this is a bad species to try to make a general comparison to a beetle for many reasons. One, there are only a few reproductives, so population dynamics will differ. Two, it has complementary sex determination with extremely high inbreeding cost due to a single gene that causes sterilization if homozygous. There are many studies on the complicated implications of its inbreeding, including as an invasive where extreme genetic load of bottlenecked populations can be weighed against reduced intraspecific aggression for e.g. supercolony formation (this is too complicated to get into here, but for more info on CSD inbreeding, see Zayed and Parker 2005, Leung and van der Meulen 2022).

Line 388: Miswritten, “have similar sibling and non-sibling mating rates”. Of course, for ants, this only applies to queens, and polyandry to reduce impacts of sib mating is possible.

Line 391: What are these other fitness costs? List them or give examples.

Line 397: The thermal idea comes out of nowhere. Is it supposed to connect to climate change and inbreeding increase? Why?

References: Formatting is inconsistent (dois for some, not forothers; capitalization varies; italics missing for species, etc).

**********

6. PLOS authors have the option to publish the peer review history of their article (what does this mean?). If published, this will include your full peer review and any attached files.

If you choose “no”, your identity will remain anonymous but your review may still be made public.

**Do you want your identity to be public for this peer review?**   For information about this choice, including consent withdrawal, please see our Privacy Policy.

Reviewer #1: No

Reviewer #2: No

**********

[NOTE: If reviewer comments were submitted as an attachment file, they will be attached to this email and accessible via the submission site. Please log into your account, locate the manuscript record, and check for the action link "View Attachments". If this link does not appear, there are no attachment files.]

To ensure your figures meet our technical requirements, please review our figure guidelines: https://journals.plos.org/plosone/s/figures

You may also use PLOS’s free figure tool, NAAS, to help you prepare publication quality figures: https://journals.plos.org/plosone/s/figures#loc-tools-for-figure-preparation.

NAAS will assess whether your figures meet our technical requirements by comparing each figure against our figure specifications.

# Author comment: Author response to Decision Letter 1

**DOI:** 10.1371/journal.pone.0335301.r002

20 Apr 2026

We have reviewed all the responses from the reviewers, addressed each and every comment in the revised manuscript with Track Changes. The responses to reviewers is also uploaded as an individual file with coloured text to indicate how and what we did to approach these suggestions. SOME LINE NUMBERS MAY HAVE BECOME MISALIGNED DUE TO USE OF DIFFERENT OPERATING SYSTEMS, we are really sorry if this is hugely changed. Let us know if there are any problems with this Line Number shifts.

We thank both the reviewers and the Editorial team for their fantastic thoughts and comments that really helped improved our study, presented here. Sincerely, R. Vasudeva

Attachment Submitted filename: PONE response to reviews final_270126.docx

# Peer review: Decision Letter 1

**DOI:** 10.1371/journal.pone.0335301.r003

**Authors:** Norman Johnson

25 May 2026

PONE-D-25-54427R1

Testing for pre- and post-copulatory inbreeding avoidance in the flour beetle Tribolium castaneum

PLOS One

Dear Dr. Vasudeva,

Thank you for submitting your manuscript to PLOS ONE. After careful consideration, we feel that it has merit but does not fully meet PLOS ONE’s publication criteria as it currently stands. Therefore, we invite you to submit a revised version of the manuscript that addresses the points raised during the review process.

Please submit your revised manuscript by Jul 09 2026 11:59PM. If you will need more time than this to complete your revisions, please reply to this message or contact the journal office at plosone@plos.org. When you're ready to submit your revision, log on to https://www.editorialmanager.com/pone/ and select the 'Submissions Needing Revision' folder to locate your manuscript file.

Please include the following items when submitting your revised manuscript:

- A letter that responds to each point raised by the academic editor and reviewer(s). You should upload this letter as a separate file labeled 'Response to Reviewers'.
- A marked-up copy of your manuscript that highlights changes made to the original version. You should upload this as a separate file labeled 'Revised Manuscript with Track Changes'.
- An unmarked version of your revised paper without tracked changes. You should upload this as a separate file labeled 'Manuscript'.

If you would like to make changes to your financial disclosure, please include your updated statement in your cover letter. Guidelines for resubmitting your figure files are available below the reviewer comments at the end of this letter.

If applicable, we recommend that you deposit your laboratory protocols in protocols.io to enhance the reproducibility of your results. Protocols.io assigns your protocol its own identifier (DOI) so that it can be cited independently in the future. For instructions see: https://journals.plos.org/plosone/s/submission-guidelines#loc-laboratory-protocols. Additionally, PLOS ONE offers an option for publishing peer-reviewed Lab Protocol articles, which describe protocols hosted on protocols.io. Read more information on sharing protocols at https://plos.org/protocols?utm_medium=editorial-email&utm_source=authorletters&utm_campaign=protocols.

As the corresponding author, your ORCID iD is verified in the submission system and will appear in the published article. PLOS supports the use of ORCID, and we encourage all coauthors to register for an ORCID iD and use it as well. Please encourage your coauthors to verify their ORCID iD within the submission system before final acceptance, as unverified ORCID iDs will not appear in the published article. *Only*   the individual author can complete the verification step; PLOS staff *cannot*   verify ORCID iDs on behalf of authors.

We look forward to receiving your revised manuscript.

Kind regards,

Norman Johnson

Academic Editor

PLOS One

Journal Requirements:

1. If the reviewer comments include a recommendation to cite specific previously published works, please review and evaluate these publications to determine whether they are relevant and should be cited. There is no requirement to cite these works unless the editor has indicated otherwise.

2. Please review your reference list to ensure that it is complete and correct. If you have cited papers that have been retracted, please include the rationale for doing so in the manuscript text, or remove these references and replace them with relevant current references. Any changes to the reference list should be mentioned in the rebuttal letter that accompanies your revised manuscript. If you need to cite a retracted article, indicate the article’s retracted status in the References list and also include a citation and full reference for the retraction notice.

Additional Editor Comments:

Both reviewers noted substantial improvement of this version compared with the previous version. One reviewer (Reviewer 1) still has substantial concerns. The most pressing concern of the reviewer (and I am in agreement) is that the authors are not fully addressing the implications of Graur and Wool (1982). This paper is not even mentioned in the Discussion and is barely mentioned elsewhere. Much more in-depth discussion of the implications of Graur and Wool is warranted in the next revision.

[Note: HTML markup is below. Please do not edit.]

Reviewer's Responses to Questions

**Comments to the Author**

1. If the authors have adequately addressed your comments raised in a previous round of review and you feel that this manuscript is now acceptable for publication, you may indicate that here to bypass the “Comments to the Author” section, enter your conflict of interest statement in the “Confidential to Editor” section, and submit your "Accept" recommendation.

Reviewer #1: (No Response)

Reviewer #2: All comments have been addressed

**********

2. Is the manuscript technically sound, and do the data support the conclusions?

The manuscript must describe a technically sound piece of scientific research with data that supports the conclusions. Experiments must have been conducted rigorously, with appropriate controls, replication, and sample sizes. The conclusions must be drawn appropriately based on the data presented.

Reviewer #1: Partly

Reviewer #2: Yes

**********

3. Has the statistical analysis been performed appropriately and rigorously?

Reviewer #1: No

Reviewer #2: I Don't Know

**********

4. Have the authors made all data underlying the findings in their manuscript fully available?

The PLOS Data policy requires authors to make all data underlying the findings described in their manuscript fully available without restriction, with rare exception (please refer to the Data Availability Statement in the manuscript PDF file). The data should be provided as part of the manuscript or its supporting information, or deposited to a public repository. For example, in addition to summary statistics, the data points behind means, medians and variance measures should be available. If there are restrictions on publicly sharing data—e.g. participant privacy or use of data from a third party—those must be specified.

Reviewer #1: Yes

Reviewer #2: Yes

**********

5. Is the manuscript presented in an intelligible fashion and written in standard English?

PLOS ONE does not copyedit accepted manuscripts, so the language in submitted articles must be clear, correct, and unambiguous. Any typographical or grammatical errors should be corrected at revision, so please note any specific errors here.

Reviewer #1: Yes

Reviewer #2: Yes

**********

6. Review Comments to the Author

Please use the space provided to explain your answers to the questions above. You may also include additional comments for the author, including concerns about dual publication, research ethics, or publication ethics. (Please upload your review as an attachment if it exceeds 20,000 characters)

Reviewer #1: I appreciate the authors’ extensive revisions and the considerable effort that went into improving the manuscript. The paper is clearer and better contextualized than the original submission, particularly with respect to the revised figures, the expanded insect literature, and the more focused Discussion. I also appreciate the authors’ willingness to engage seriously with my initial comments.

That said, I remain unconvinced by the authors’ treatment of the Graur and Wool (1982) issue, which I still view as central to interpretation of the assortative mating results.

My concern was not simply whether rearing treatment and mating outcome are statistically associated in a pooled 2×4 contingency table. Rather, the biologically relevant observation from Graur and Wool is that assortative mating was observed under one rearing regime (“reared apart”) and disappeared under the other (“reared together”). The present study effectively reproduces only the “reared apart” condition. Therefore, the key interpretive limitation remains: it is unclear whether the observed sibling preference reflects kin recognition, familiarity effects, or an artifact of the isolation-rearing design. The lack of significance in the pooled association test does not negate the observation that assortative mating was present in one treatment and absent in the other. As written, the response reads as though the authors are attempting to statistically dismiss a biologically meaningful pattern rather than acknowledge it as an important caveat for the interpretation of their own findings.

I also do not find the authors’ argument persuasive that isolation is the “natural” design because beetles in nature would encounter mixed-relatedness groups. If anything, this seems to strengthen the rationale for testing beetles under mixed-rearing conditions rather than isolated rearing. If the goal is to infer mate choice under natural social conditions, rearing individuals in complete isolation may remove developmental and social cues that are themselves biologically important to mate recognition. Thus, I still think the manuscript should frame the assortative mating result much more cautiously and explicitly acknowledge the limitations imposed by the rearing design.

There is also a concerning error in the rebuttal text immediately following the contingency-table discussion, where Tribolium are mistakenly referred to as “crickets.” While obviously minor in isolation, it contributes to a broader impression of the work.

I continue to think the study contains meaningful results that are consistent with previous work in the system especially regarding offspring viability and sperm precedence dynamics, and the manuscript is improved overall. However, I still believe the conclusions regarding assortative mating need to be substantially tempered. The final two paragraphs of their conclusions rest on having shown that Tribolium demonstrate positive assortative mating, but I don't think their designs allows them to make this conclusion outside the very narrow scope of their rearing/breeding design, and consequently the attempts to explain it are moot.

Reviewer #2: The manuscript is much improved in its use of updated literature and corrections of various small errors, unclear language, and unsubstantiated discussion points (thermal stress). It is to be commended for clear and effective counter points for all concerns across all reviewers. I give minor comments below, but otherwise recommend acceptance.

Line 30: unclear what by “insects without parental care,” does not connect to the idea of with multiple mating

Line 34: Better phrased as “direct observation of breeding behavior and subsequent scoring of offspring parentage using phenotypic markers”

Line 37: Offspring of unrelated parents?

Line 38: Not clear what “raw” count is

Line 40: Fertilisation success of male with related and unrelated

Line 42: This result is unclear. Did total number of offspring even out between 1st and 2nd male over the 5 weeks, or did new offspring after week 5 have equal percentage paternity?

Line 49: Homozygosity across the whole genome, one gene, or?

Line 59: Make clear that this is for mate choice.

Line 75: Studies in Drosophila failed to find any evidence for potential pre-or post-copulatory inbreeding avoidance.

Line 97: Missing word after “P2”? What do the more attractive males have more of?

Line 105: “mated to”

Line 107: Italicize Tribolium castaneum

Line 265: Here the meaning of P2 becomes clear, but please make it clear in abstract and earlier. P2 instinctively sounds like a parental generation notation.

Figure 2B: It is still not clear to me what is meant about the 2nd male effect resulting in equal performance between the sib and non-sib. From this figure, I think the conclusion is drawn from pooling data of each type of treatment across data points for multiple weeks, not a reversed trend at some point. Please make this clear in wording throughout.

Line 340: I think the result of preferential sibling mating is significant, but that the effect size isn’t very big (with the exception of the female mate choice for mating attempts? But the difference is something like 2 vs 4, and there is a lot of scatter for the sib mating data; Figure 1B). I think it better to be transparent about this, as it is still of great importance and strong statement that there isn’t major inbreeding avoidance. I am not sure if the authors want to be emphatic about evidence for sibling preference.

Line 349: In the same vein, do authors want to take such a soft stance on inbreeding depression? In figure 1D, there is definitely a cost to offspring viability with sib mating that doesn’t seem that small (~25%). Coming to a paradoxical conclusion of some sibling preference/no sibling avoidance despite offspring depression, would also be valid. There could be hidden benefits (e.g. with the authors’ suggestion of inclusive fitness), but it could also just be maladaptive.

Line 357: Not sure what is meant by highly polymorphic genes. Siblings would have the same alleles and perceive them via phenotype? Perhaps the idea of phenotype matching here makes sense, as in

Mathieu Lihoreau, Cédric Zimmer, Colette Rivault, Kin recognition and incest avoidance in a group-living insect, Behavioral Ecology, Volume 18, Issue 5, September 2007, Pages 880–887, https://doi.org/10.1093/beheco/arm046

Or speculation on recognizing olfactory cues e.g. as with sibling recognition in social species

Couto, A., Marty, S., Dawson, E.H., d'Ettorre, P., Sandoz, J.-C. and Montgomery, S.H. (2023), Evolution of the neuronal substrate for kin recognition in social Hymenoptera. Biol Rev, 98: 2226-2242. https://doi.org/10.1111/brv.13003

Line 390-392: There is a rough transition here of going from saying inbreeding avoidance isn’t as prevalent as previously thought into a big inbreeding avoidance example. Needs a phrase like: “There are clear cases where inbreeding avoidance is employed; for example, crickets”… and then it switches over naturally to the inbreeding prone species. I think there are also good examples from bedbugs and parasitoids where sib mating is the rule.

**********

7. PLOS authors have the option to publish the peer review history of their article (what does this mean?). If published, this will include your full peer review and any attached files.

If you choose “no”, your identity will remain anonymous but your review may still be made public.

**Do you want your identity to be public for this peer review?**   For information about this choice, including consent withdrawal, please see our Privacy Policy.

Reviewer #1: No

Reviewer #2: No

**********

[NOTE: If reviewer comments were submitted as an attachment file, they will be attached to this email and accessible via the submission site. Please log into your account, locate the manuscript record, and check for the action link "View Attachments". If this link does not appear, there are no attachment files.]

To ensure your figures meet our technical requirements, please review our figure guidelines: https://journals.plos.org/plosone/s/figures

You may also use PLOS’s free figure tool, NAAS, to help you prepare publication quality figures: https://journals.plos.org/plosone/s/figures#loc-tools-for-figure-preparation.

NAAS will assess whether your figures meet our technical requirements by comparing each figure against our figure specifications.

# Author comment: Author response to Decision Letter 2

**DOI:** 10.1371/journal.pone.0335301.r004

3 Jun 2026

Dear Norman,

Thank you for your positive feedback on our revised manuscript and that of the reviewers. We have followed the reviewers most recent suggestions in relation to the ms as detailed below.

Reviewer #1: I appreciate the authors’ extensive revisions and the considerable effort that went into improving the manuscript. The paper is clearer and better contextualized than the original submission, particularly with respect to the revised figures, the expanded insect literature, and the more focused Discussion. I also appreciate the authors’ willingness to engage seriously with my initial comments.

Thank you.

That said, I remain unconvinced by the authors’ treatment of the Graur and Wool (1982) issue, which I still view as central to interpretation of the assortative mating results. My concern was not simply whether rearing treatment and mating outcome are statistically associated in a pooled 2×4 contingency table. Rather, the biologically relevant observation from Graur and Wool is that assortative mating was observed under one rearing regime (“reared apart”) and disappeared under the other (“reared together”). The present study effectively reproduces only the “reared apart” condition. Therefore, the key interpretive limitation remains: it is unclear whether the observed sibling preference reflects kin recognition, familiarity effects, or an artifact of the isolation-rearing design. The lack of significance in the pooled association test does not negate the observation that assortative mating was present in one treatment and absent in the other. As written, the response reads as though the authors are attempting to statistically dismiss a biologically meaningful pattern rather than acknowledge it as an important caveat for the interpretation of their own findings.

I also do not find the authors’ argument persuasive that isolation is the “natural” design because beetles in nature would encounter mixed-relatedness groups. If anything, this seems to strengthen the rationale for testing beetles under mixed-rearing conditions rather than isolated rearing. If the goal is to infer mate choice under natural social conditions, rearing individuals in complete isolation may remove developmental and social cues that are themselves biologically important to mate recognition. Thus, I still think the manuscript should frame the assortative mating result much more cautiously and explicitly acknowledge the limitations imposed by the rearing design.

The reason for our statistical analysis was to determine whether the result in Graur and Wool is likely to be biologically meaningful or could simply be a result of chance. That analysis shows that there may actually be no effect of rearing alone or apart, but it was not designed to ‘get us off the hook’ in relation to this point. We take the reviewer’s point that even if there is only very weak evidence (or indeed, no evidence) that rearing apart as opposed to in a group has an effect, it remains possible that it does. We have added a new paragraph to the discussion where we discuss this point. We have added new references to studies of other insects that show the potential for the type of effect that the reviewer is referring to. We have borrowed the reviewer’s specific wording that “rearing individuals in complete isolation may remove developmental and social cues that are themselves biologically important to mate recognition” (L361-362)– we agree with the thrust of this point. We think with this new discussion readers will be fully aware of the issues the reviewer is referring to.

There is also a concerning error in the rebuttal text immediately following the contingency-table discussion, where Tribolium are mistakenly referred to as “crickets.” While obviously minor in isolation, it contributes to a broader impression of the work.

Yes, apologies for this careless typo in our letter.

I continue to think the study contains meaningful results that are consistent with previous work in the system especially regarding offspring viability and sperm precedence dynamics, and the manuscript is improved overall. However, I still believe the conclusions regarding assortative mating need to be substantially tempered. The final two paragraphs of their conclusions rest on having shown that Tribolium demonstrate positive assortative mating, but I don't think their designs allows them to make this conclusion outside the very narrow scope of their rearing/breeding design, and consequently the attempts to explain it are moot.

We have revised our text so that we repeatedly point out that our conclusions can only be very confidently ascribed to the rearing situation that we employed (e.g., L363-365).

Reviewer #2: The manuscript is much improved in its use of updated literature and corrections of various small errors, unclear language, and unsubstantiated discussion points (thermal stress). It is to be commended for clear and effective counter points for all concerns across all reviewers. I give minor comments below, but otherwise recommend acceptance.

Thank you

Line 30: unclear what by “insects without parental care,” does not connect to the idea of with multiple mating

Yes, fair point we have taken out that clause as it either needs a lot more explanation or needs to be left out, which makes more sense.

Line 34: Better phrased as “direct observation of breeding behavior and subsequent scoring of offspring parentage using phenotypic markers”

Thank you we have used the suggested text

Line 37: Offspring of unrelated parents?

Yes we have revised this sentence in line with this suggestion.

Line 38: Not clear what “raw” count is

Yes, good point, we have removed the confusing reference to ‘raw count’.

Line 40: Fertilisation success of male with related and unrelated

We have revised this sentence

Line 42: This result is unclear. Did total number of offspring even out between 1st and 2nd male over the 5 weeks, or did new offspring after week 5 have equal percentage paternity?

This is a very good point; we have revised the text to clarify that it is the latter scenario.

Line 49: Homozygosity across the whole genome, one gene, or?

We have added ‘across the genome’.

Line 59: Make clear that this is for mate choice.

Done

Line 75: Studies in Drosophila failed to find any evidence for potential pre-or post-copulatory inbreeding avoidance.

Done

Line 97: Missing word after “P2”? What do the more attractive males have more of?

We have removed the term P2 from the abstract in relation to the suggestion made below.

Line 105: “mated to”

Done

Line 107: Italicize Tribolium castaneum

Done

Line 265: Here the meaning of P2 becomes clear, but please make it clear in abstract and earlier. P2 instinctively sounds like a parental generation notation.

Apologies – it is a good point that the term P2 will not be familiar to all readers. We have removed it from the abstract and explain it fully the first time we use it in the revised manuscript (L98-99).

Figure 2B: It is still not clear to me what is meant about the 2nd male effect resulting in equal performance between the sib and non-sib. From this figure, I think the conclusion is drawn from pooling data of each type of treatment across data points for multiple weeks, not a reversed trend at some point. Please make this clear in wording throughout.

It is not the 2nd male effect that results in equal performance, it is adjusting for the fact that offspring from sibling males are less likely to survive (developmental failures) so their fertilisation success is underestimated if we just use the raw number of offspring they sire. It is clear that our text was not clear enough on this point, we have revised the figure legend to clarify this point where it is most important (L265-250).

Line 340: I think the result of preferential sibling mating is significant, but that the effect size isn’t very big (with the exception of the female mate choice for mating attempts? But the difference is something like 2 vs 4, and there is a lot of scatter for the sib mating data; Figure 1B). I think it better to be transparent about this, as it is still of great importance and strong statement that there isn’t major inbreeding avoidance. I am not sure if the authors want to be emphatic about evidence for sibling preference.

Yes, this is a fair point. We have revised the text so that it reads that “Males placed with a sibling and a non-sibling female attempted to mate with their sisters and actually succeeded in mating with them slightly more often than unrelated females.” (L345-347) Adding the word ‘slightly’ is a small addition, but we think it does convey the lack of being emphatic that the reviewer is requesting.

Line 349: In the same vein, do authors want to take such a soft stance on inbreeding depression? In figure 1D, there is definitely a cost to offspring viability with sib mating that doesn’t seem that small (~25%). Coming to a paradoxical conclusion of some sibling preference/no sibling avoidance despite offspring depression, would also be valid. There could be hidden benefits (e.g. with the authors’ suggestion of inclusive fitness), but it could also just be maladaptive.

We do point out the costs of inbreeding are large. However, as shown in the models we cite, the inclusive fitness benefits of sib mating can be large, and this is reflected in our text (L355-369). It could simply be maladaptive, but we feel that this is something that readers will be aware of, and adding a caveat of that type is more likely to puzzle readers than to enlighten them.

Line 357: Not sure what is meant by highly polymorphic genes. Siblings would have the same alleles and perceive them via phenotype? Perhaps the idea of phenotype matching here makes sense, as in

Mathieu Lihoreau, Cédric Zimmer, Colette Rivault, Kin recognition and incest avoidance in a group-living insect, Behavioral Ecology, Volume 18, Issue 5, September 2007, Pages 880–887, https://doi.org/10.1093/beheco/arm046

Or speculation on recognizing olfactory cues e.g. as with sibling recognition in social species

Couto, A., Marty, S., Dawson, E.H., d'Ettorre, P., Sandoz, J.-C. and Montgomery, S.H. (2023), Evolution of the neuronal substrate for kin recognition in social Hymenoptera. Biol Rev, 98: 2226-2242. https://doi.org/10.1111/brv.13003

Thank you – we clarified our point (which is as the reviewer suggests) and added the 1st reference suggested above.

Line 390-392: There is a rough transition here of going from saying inbreeding avoidance isn’t as prevalent as previously thought into a big inbreeding avoidance example. Needs a phrase like: “There are clear cases where inbreeding avoidance is employed; for example, crickets”… and then it switches over naturally to the inbreeding prone species. I think there are also good examples from bedbugs and parasitoids where sib mating is the rule.

Thank you we included the suggested text in the revised manuscript.

Attachment Submitted filename: PONE 2nd response to reviewers.docx

# Editor report: Decision Letter 2

**DOI:** 10.1371/journal.pone.0335301.r005

**Authors:** Norman Johnson

5 Jun 2026

Testing for pre- and post-copulatory inbreeding avoidance in the flour beetle Tribolium castaneum

PONE-D-25-54427R2

Dear Dr. Vasudeva,

We’re pleased to inform you that your manuscript has been judged scientifically suitable for publication and will be formally accepted for publication once it meets all outstanding technical requirements.

Within one week, you’ll receive an e-mail detailing the required amendments. When these have been addressed, you’ll receive a formal acceptance letter and your manuscript will be scheduled for publication.

An invoice will be generated when your article is formally accepted. Please note, if your institution has a publishing partnership with PLOS and your article meets the relevant criteria, all or part of your publication costs will be covered. Please make sure your user information is up-to-date by logging into Editorial Manager at Editorial Manager® and clicking the ‘Update My Information' link at the top of the page. For questions related to billing, please contact billing support.

If your institution or institutions have a press office, please notify them about your upcoming paper to help maximize its impact. If they’ll be preparing press materials, please inform our press team as soon as possible -- no later than 48 hours after receiving the formal acceptance. Your manuscript will remain under strict press embargo until 2 pm Eastern Time on the date of publication. For more information, please contact onepress@plos.org.

Kind regards,

Norman Johnson

Academic Editor

PLOS One

Additional Editor Comments (optional):

The authors have addressed the concerns of the reviewers to my satisfaction. Notably, the second paragraph of the Discussion addresses a limitation of the study related to the previous Graur and Wool study.

Reviewers' comments:

# Editor report: Acceptance letter

**DOI:** 10.1371/journal.pone.0335301.r006

**Authors:** Norman Johnson

PONE-D-25-54427R2

PLOS One

Dear Dr. Vasudeva,

I'm pleased to inform you that your manuscript has been deemed suitable for publication in PLOS One. Congratulations! Your manuscript is now being handed over to our production team.

At this stage, our production department will prepare your paper for publication. This includes ensuring the following:

* All references, tables, and figures are properly cited

* All relevant supporting information is included in the manuscript submission,

* There are no issues that prevent the paper from being properly typeset

You will receive further instructions from the production team, including instructions on how to review your proof when it is ready. Please keep in mind that we are working through a large volume of accepted articles, so please give us a few days to review your paper and let you know the next and final steps.

Lastly, if your institution or institutions have a press office, please let them know about your upcoming paper now to help maximize its impact. If they'll be preparing press materials, please inform our press team within the next 48 hours. Your manuscript will remain under strict press embargo until 2 pm Eastern Time on the date of publication. For more information, please contact onepress@plos.org.

You will receive an invoice from PLOS for your publication fee after your manuscript has reached the completed accept phase. If you receive an email requesting payment before acceptance or for any other service, this may be a phishing scheme. Learn how to identify phishing emails and protect your accounts at https://explore.plos.org/phishing.

If we can help with anything else, please email us at customercare@plos.org.

Thank you for submitting your work to PLOS ONE and supporting open access.

Kind regards,

PLOS ONE Editorial Office Staff

on behalf of

Dr. Norman Johnson

Academic Editor

PLOS One
